# Simultaneous Prediction of Wheat Yield and Grain Protein Content Using Multitask Deep Learning from Time-Series Proximal Sensing

**DOI:** 10.34133/2022/9757948

**Published:** 2022-03-29

**Authors:** Zhuangzhuang Sun, Qing Li, Shichao Jin, Yunlin Song, Shan Xu, Xiao Wang, Jian Cai, Qin Zhou, Yan Ge, Ruinan Zhang, Jingrong Zang, Dong Jiang

**Affiliations:** ^1^Plant Phenomics Research Centre, Academy for Advanced Interdisciplinary Studies, Regional Technique Innovation Center for Wheat Production, Ministry of Agriculture, Key Laboratory of Crop Physiology and Ecology in Southern China, Ministry of Agriculture, Collaborative Innovation Centre for Modern Crop Production Co-Sponsored by Province and Ministry, Nanjing Agricultural University, Nanjing 210095, China; ^2^Jiangsu Provincial Key Laboratory of Geographic Information Science and Technology, International Institute for Earth System Sciences, Nanjing University, Nanjing, Jiangsu 210023, China

## Abstract

Wheat yield and grain protein content (GPC) are two main optimization targets for breeding and cultivation. Remote sensing provides nondestructive and early predictions of yield and GPC, respectively. However, whether it is possible to simultaneously predict yield and GPC in one model and the accuracy and influencing factors are still unclear. In this study, we made a systematic comparison of different deep learning models in terms of data fusion, time-series feature extraction, and multitask learning. The results showed that time-series data fusion significantly improved yield and GPC prediction accuracy with *R*^2^ values of 0.817 and 0.809. Multitask learning achieved simultaneous prediction of yield and GPC with comparable accuracy to the single-task model. We further proposed a two-to-two model that combines data fusion (two kinds of data sources for input) and multitask learning (two outputs) and compared different feature extraction layers, including RNN (recurrent neural network), LSTM (long short-term memory), CNN (convolutional neural network), and attention module. The two-to-two model with the attention module achieved the best prediction accuracy for yield (*R*^2^ = 0.833) and GPC (*R*^2^ = 0.846). The temporal distribution of feature importance was visualized based on the attention feature values. Although the temporal patterns of structural traits and spectral traits were inconsistent, the overall importance of both structural traits and spectral traits at the postanthesis stage was more important than that at the preanthesis stage. This study provides new insights into the simultaneous prediction of yield and GPC using deep learning from time-series proximal sensing, which may contribute to the accurate and efficient predictions of agricultural production.

## 1. Introduction

Wheat yield and grain protein content (GPC) are two main optimizing target traits for breeding and cultivation to ensure food security [1] and improve food quality [2] under the pressure of ever-increasing world populations and living standards [3]. Previous studies have focused on yield prediction, while quality assessment has received unprecedented attention with the improvement of people's living standards in recent years. Therefore, simultaneous prediction of yield and GPC is increasingly important [4] to meet the requirement of simultaneous selection of high yield and GPC varieties.

Remote sensing has become an important data source for yield and protein prediction in previous decades [5]. Yu et al. [6] predicted soybean yield (*r* = 0.82) using the random forest method from UAV-based high-resolution multispectral data. Grain yield and GPC have also been successfully predicted for maize [7], wheat ([8]; Li et al., [9, 10]), and barley [11]. In these models, yield and protein content are predicted separately by establishing different machine learning models. Due to the intrinsic relationship between yield and protein content [5, 12], whether it is possible to build a model to predict yield and quality simultaneously is worth exploring.

Recent studies emphasize the need to fuse multisource and multitemporal data for remote sensing-based predictions of yield and GPC. Multisource data enrich knowledge of feature dimensions, such as structural and spectral traits. Combining the characteristics of multisource information is beneficial to complement each other and improve prediction accuracy, as reported in potato [9, 10], soybean [13], and cotton [14]. In addition, multitemporal data usually outperform single-stage data due to the cumulative information in the time dimension. The cumulative vegetation index substantially outperforms the single-stage vegetation index for yield estimation [15], and the best time interval is usually from the jointing to the initial filling stage [8]. However, these studies only fused data of several stages, such as standing, jointing, heading, and filling growth stages. Time-series (e.g., daily) data are more common in field phenotyping studies with the recent prosperity of proximal sensing. It is challenging to fuse data from the entire growth stage in these machine learning methods.

Deep learning, a branch of the data-driven machine learning method, has been proven effective in dealing with large-volume, high-dimensional, time-series data and solving multiple tasks simultaneously [16, 17]. Through multilayer neural network connections, automatic learning of nonlinear features, and optimizing massive parameters, deep learning shows innate strengths in solving big data and high-dimensional complex problems [18]. To improve time-series feature extraction and target prediction, models with memory capabilities such as RNN (recurrent neural network) and LSTM (long short-term memory) have been proposed and widely used [19]. To achieve simultaneous learning of multiple tasks, multitask learning has been developed, which can improve the accuracy and overall speed of several related deep learning tasks through associative training [20]. Since deep learning was proposed, it has achieved state-of-the-art results in many fields (e.g., image processing) [21], but it has not yet become prevailing for prediction in agriculture.

In yield prediction, Maimaitijiang et al. [13] found that deep learning-based models are better than traditional machine learning methods. In addition, they found that data fusion (RGB, multispectral, and thermal) also improved the performance of deep learning. Sandhu et al. [22] also proved that the yield prediction accuracy of the deep learning model was 0 to 5% higher than that of a ridge regression model for predicting complex traits. They also pointed out that MLP (multilayer perceptron) produced higher prediction accuracy than CNN (convolutional neural network). These efforts proved the effectiveness of deep learning [23]. However, deep learning for yield and GPC prediction is still in its infancy, posing many questions to be explored that are related to data fusion, time-series feature extraction, and multitask learning (e.g., simultaneous prediction of yield and GPC).

This study collected near-daily multispectral and LiDAR data during the whole crop growth cycle from a high-throughput phenotyping platform. The aims of this study include the following: (1) to explore the benefits of data fusion, multitask learning, and feature extraction strategies to the yield and GPC prediction accuracy of deep learning-based models; (2) to propose a new deep learning model by fusing the benefits of multimodal data, an optimal feature extraction module, and multitask learning; and (3) to illustrate the time-series feature contribution by visualizing features of a temporal-channel attention layer.

## 2. Study Area and Data Collection

### 2.1. Experimental Design

**Figure 1:**
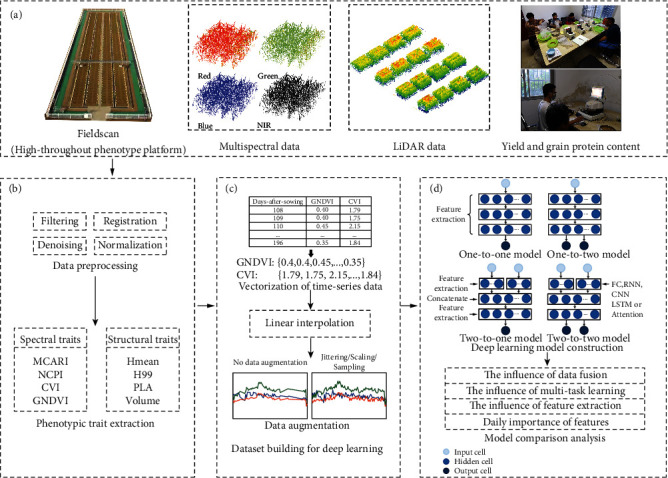
A workflow diagram of the experimental design, feature extraction, and modeling. (a) Study area and data collection; (b) data preprocessing for original data and phenotypic trait extraction; (c) dataset building for deep learning and model construction; and (d) deep learning model construction and model comparison analysis.

The experiment was conducted at the Baima Experimental Station (119°18′71^″^E, 31°62′00^″^N) of Nanjing Agricultural University, China (Figure 1(a)). The area of the planting field is approximately 2000 m^2^. The field was split into 480 plots, which were composed of four blocks that contained two nitrogen (N) fertilizer levels (control group, 240 kg/ha; N deficiency group, 0 kg/ha) and two replications according to the principle of split-plot design. A total of 120 Chinese winter wheat varieties with various levels of grain yield and protein potential were selected and planted in each replication. The plot size was 1 × 1 m, the row spacing was 0.25 m, the plot spacing was 0.5 m, and the germplasm density was 300 seeds/m^2^.

Soil samples were collected to confirm that the soil basic nitrogen content was sufficiently low for the requirement of our designed nitrogen treatments from the plow layer before sowing. The total organic nitrogen content (0.67 g/kg) is much lower than that in normal soil and even lower than that in poor soil (0.98 g/kg) [24]. Nitrogen fertilizer (urea, 46%) was applied twice for the control group, half before sowing and the remaining half at the jointing stage. The quantities of phosphorus (P_2_O_5_, 12%) and potassium (K_2_O, 60%) fertilizer in the two treatments were both 120 kg/ha and applied as the base fertilizer before sowing. All plots were manually planted in November 2019 and harvested in May 2020, with careful field management throughout the whole growth period.

### 2.2. Grain Yield and Protein Content Collection

The grain yield and protein content data were collected manually for all plots at the mature stage (Figure 1(a)), taking approximately two months. In each plot, all aboveground portions were manually harvested. Then, the grains of each plot were manually threshed and filtered from the spikes, which were then dehydrated under sunlight, weighed, and recorded as yield (kg ha^−1^). Then, a subsample of grains from each plot was extracted and poured into a plastic sample tray, and the grain protein content (%) was measured using a NIR DA7250TM NIR analyzer (*Perten Instruments, Inc., IL, USA*).

### 2.3. Proximal Sensing Data Collection

This study collected both three-dimensional (3D) LiDAR and multispectral data of each plot using a high-throughput phenotyping platform (FieldScan) [25] (Figure 1(a)). The FieldScan is equipped with two groups of sensor suites to speed up data collection. Each group has two sensor suits installed at a certain angle according to the field of view to enhance the ability of 3D data acquisition. The sensor suite is named PlantEye F500 (Phenospex, Heerlen, Netherlands), which is composed of one near-infrared laser scanning sensor (LiDAR) and one four-band spectral sensor (multispectral).

LiDAR and multispectral data are collected simultaneously with the platform moving at a speed of 5 cm/s. The point resolutions are 0.8, 0.8, and 0.2 mm in the *xyz* direction. The multispectral data include blue (460-485 nm, B), green (530-540 nm, G), red (620-645 nm, R), and near-infrared (720-750 nm, NIR) wavelengths. The multispectral data were collected under a stable artificial light environment. The FieldScan platform worked four times near-daily from the tillering stage to the maturity stage (107 days after sowing to 195 days after sowing, Supplementary Table S2), accumulating approximately 2 TB of data. In this study, data collected at night were selected for analysis to avoid the unnecessary influence of environmental wind and light.

## 3. Method

### 3.1. Data Preprocessing

The point cloud data collected by two laser scanners in each group were registered to increase the point cloud density (approximately 800000 pts/m^3^) using the commercial web-based interface software system (HortControl). The software also fused multispectral information with each point automatically, making all points contain both geometric information (i.e., *xyz* coordinates) and spectral information (i.e., R, G, B, and NIR). After data registration and fusion, the fused points were processed with filtering, denoising, and normalization, as described in [26]. The normalized data were used for extracting phenotypic traits of each plot, including structural traits and spectral traits (Figure 1(b)).

### 3.2. Phenotypic Trait Extraction

Eight phenotypic traits were selected and extracted from the fused points due to their wide application in agricultural practice [7, 27, 28]. Among them, four spectral traits have been widely used to estimate yield and protein content in crops [8, 13], including the green normalized difference vegetation index (GNDVI) [15], the chlorophyll vegetation index (CVI) [29], the normalized chlorophyll pigment vegetation index (NCPI) [30], and the modified chlorophyll absorption in reflectance index (MCARI) [31]. GNDVI is insensitive to plant structure variation and is correlated with yield better than the normalized difference vegetation index (NDVI) [15, 32]. The CVI is sensitive to plant chlorophyll concentration [29]. NCPI can estimate the proportion of total photosynthetic pigments to chlorophyll, especially under nitrogen deficiency conditions [30]. MCARI is modified from the chlorophyll absorption in reflectance index (CARI) to minimize the nonplant effects on spectral reflectance [31]. These spectral traits were calculated based on the spectral attributes of the points (Table S1), and their dynamics are shown in Figure S1.

The other four structural traits are the mean height of points (Hmean), 99 percentile height of points (H99), plant volume (volume), and projected leaf area (PLA). Hmean and H99 present the mean and 99% percentile height of all points, respectively [33]. PLA and volume are both highly correlated with plant productivity [28, 34]. These structural traits were extracted from the fused point cloud data based on geometric information using the methods in Jin et al. [26], and their temporal dynamics are shown in Figure S1.

### 3.3. Dataset Preparation for Deep Learning

Deep learning, a data-driven machine learning method, requires a large amount of regular data to enable automatic feature extraction and batch learning. The original data need to be organized into a unified structure (e.g., unified spatial dimension and time continuity of data). In addition, data augmentation has been proven useful for improving model accuracy. Therefore, our dataset was built by the following steps, including data vectorization, missing value interpolation, data augmentation, and data normalization.

Data vectorization was conducted to organize the extracted phenotypic traits of each plot into unified feature vectors. Values of each phenotypic trait extracted from 107 days after sowing (DAS) to 195 DAS were concatenated into a one-dimensional (1D) feature vector {*x*_107_, *x*_108_, *x*_109_, *x*_110_, ⋯, *x*_195_}. For example, *x*_107_ represents the trait value on the 107^th^ day after seeding.

Missing value interpolation was implemented to fill the missing values in the original data. There were some missing values due to some irresistible factors, such as equipment maintenance and power outages. Because the missing values of each plot vary during the whole growth stage, the lengths of inputs are too different to be trained in batch. A linear interpolation method was used to address the missing values.

Data augmentation can generate more diverse data, which has been proven useful for improving the generalization ability and preventing overfitting of a deep learning model. Based on the 480 examples, jittering, scaling, and random sampling methods were adopted to augment the data by referring to previous studies [1]. Given an example, one of the methods (jittering, scaling, and random sampling) was randomly applied to its feature vector values, and its target values (yield and GPC) were added by the value generated from a normal distribution between -0.05 and 0.05. In this way, the total example size was increased from 480 to 1000 (Table 1).

Data normalization was beneficial to model accuracy and coverage speed [35]. In this study, min-max normalization was applied to transform the values of the yield and GPC to the interval [0, 1].

### 3.4. Deep Learning Model Construction and Validation

#### 3.4.1. Model Structure

**Figure 2:**
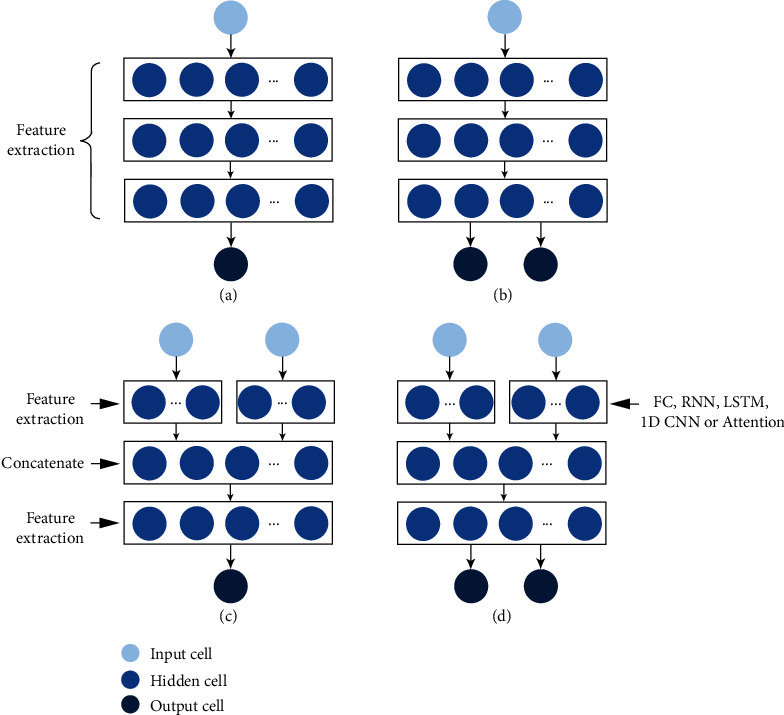
Model structures. (a) One-to-one model; (b) one-to-two model; (c) two-to-one model; and (d) two-to-two model.

To verify the effectiveness of data fusion and multitask learning, four fully connected (FC) neural network structures were proposed according to the input feature types and output variables of each model, including a one-to-one model, a one-to-two model, a two-to-one model, and a two-to-two model (Figure 2).

The one-to-one model was employed to compare the predictive performance using either one structural trait or one spectral trait. The architecture of the one-to-one model was an FC neural network with one input layer, three hidden layers, and one output layer (Figure 2(a)). The input is one trait, and the output is the predicted yield or GPC. Three hidden layers were used for feature extraction, and the number of neurons in each hidden layer was 16. More details of this model structure are shown in Figure S2(a). The loss function of this model is the mean absolute error (MAE) between the prediction and ground truth value.

The one-to-two model was designed for multitask learning. The architecture includes one input layer, three hidden layers, and two output layers (Figure 2(b)). The input is also one spectral trait or one structural trait, while the output layers predict both yield and GPC. Details of this model structure are shown in Figure S2(b). Since the model has two output variables, the loss function of this model is designed as the weighted value of yield MAE and GPC MAE (Eq. (1)).

(1) $$\begin{array}{c} \text{Total Loss}=\lambda 1\times {\text{MAE}}_{\text{GPC}}+\lambda 2\times {\text{MAE}}_{\text{Yield}}, \end{array}$$

where *λ*1 and *λ*2 represent the weight values of GPC and yield loss, respectively. The default values of *λ*1 and *λ*2 were both 0.5, giving the same weight to the two tasks. The influences of setting the default ratio are discussed in Section 5.3.

The two-to-one model was designed for data fusion at the feature level according to previous findings that intermedian-level fusion performs better than input-level data fusion [13]. The architecture consists of two input layers, three hidden layers, and one output layer (Figure 2(c)). The input layer contains two parts, one for loading data from spectral traits and the other for structural traits. The input dimensions of the left and right parts are both 1 × 89, which are transformed into 1 × 16 by one hidden layer for initial feature extraction. Then, a concatenate layer fuses these two feature vectors (1 × 16) into a 1 × 32 vector. The last hidden layer transforms the fused feature vector into a 1 × 16 vector for further feature extraction. The loss function of this model is the mean absolute error (MAE). More details of this model structure are shown in Figure S2(c).

The two-to-two model was designed to integrate data fusion and multitask learning. The architecture consists of two input layers, three hidden layers, and two output layers. The input layers and hidden layers are the same as the two-to-one model, and the loss function and output layers are the same as the one-to-two model. To better extract useful features from time-series data, RNN, LSTM, 1D CNN, and attention mechanism module were used to replace the default FC in the initial feature extraction. RNN, LSTM, and 1D CNN were implemented with built-in functions in Keras. The attention mechanism module is designed manually: (1) The dimension of the original input is transformed from 1 × 89 × 1 into 1 × 89 × 5 by one LSTM layer for initial feature extraction; (2) two parallel network lines are designed: one line preserves the initial feature information (1 × 89 × 5), and the other is permuted twice to achieve attention weight values of the initial feature (1 × 89 × 5); and (3) the above two feature matrices are multiplied (1 × 89 × 5). The loss function of the two-to-two model is the same as that of the one-to-two model. Details of all two-to-two model structures are shown in Figure S2(d)-(h). All models were implemented using Python with TensorFlow and Keras libraries.

#### 3.4.2. Model Training

The dataset was randomly split into a training dataset, validation dataset, and testing dataset at ratios of 80%, 10%, and 10%, respectively. All models were trained on a high-performance computer with an Intel i7 8700 central processing unit (CPU), 32 GB memory, and an NVIDIA 2080 GeForce graphics processing unit (GPU). The batch size was 32. The Adam method with a learning rate of 0.001 was set to optimize the model parameters. The Dropout regularization method with a rate of 0.1 was used to reduce overfitting due to its flexibility and robustness [36]. All models were trained to converge with the early stopping strategy in Keras.

#### 3.4.3. Model Validation

To evaluate the performance of the prediction model, the coefficients of determination (*R*^2^), root mean square error (RMSE), and relative RMSE (rRMSE) were calculated using the following formulas.

(2) $$\begin{array}{c} R^{2}=1-\frac{\sum_{i}^{n}{\left(yi-\overset{⌢}{y}i\right)}^{2}}{\sum_{i}^{n}{\left(yi-\bar{y}i\right)}^{2}}, \end{array}$$

(3) $$\begin{array}{c} \text{RMSE}=\sqrt{\frac{\sum_{i=1}^{n}{\left(yi-\overset{⌢}{y}i\right)}^{2}}{n}}, \end{array}$$

(4) $$\begin{array}{c} r\text{RMSE}=\frac{\text{RMSE}}{\bar{y}}*100\%, \end{array}$$

where *yi* and $\overset{⌢}{y}i$ are the measured and predicted values, respectively. $\bar{y}$ is the mean of the measured values, and *n* is the total number of examples in the testing dataset.

### 3.5. Model Analysis

The model comparison analysis includes two parts, one for analyzing data fusion, multitask, and different feature extraction methods on the prediction accuracies. The other part is building an optimal architecture for the simultaneous prediction of wheat yield and GPC, as well as analyzing the daily importance of features for model prediction.

To select the optimal spectral traits and structural traits, each trait was input into a one-to-one model in turn to assess the importance of each trait. The best prediction results of the one-to-one model were used as the benchmark for comparing the performances of other types of models (two-to-one, one-to-two, and two-to-two models). To verify the effectiveness of data fusion and multitask learning, a two-to-one model for data fusion and a one-to-two model for multitask learning are constructed and compared with the best one-to-one model. The inputs of the two-to-one model are the best spectral trait and the best structural trait, according to the results of the one-to-one model. Finally, the performances of various two-to-two models using different feature extraction methods, including RNN, LSTM, CNN, and attention mechanism module, are compared with the simple FC two-to-two model. According to the analysis of data fusion, multitask learning, and feature extraction, the best model was proposed. Additionally, the daily importance of predictors is analyzed using the attention feature.

## 4. Results

### 4.1. One-to-One Model Performance in Yield and GPC Prediction

**Figure 3:**
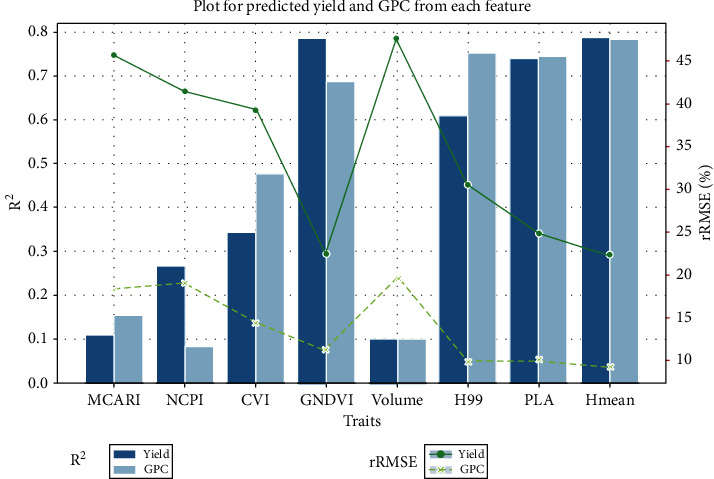
Prediction performance comparison among different traits for yield and GPC using a one-to-one model.

Spectral traits (MCARI, NCPI, CVI, and GNDVI) and structural traits (Volume, H99, PLA, and Hmean) were applied to predict wheat yield and GPC based on the one-to-one model (Figure 3). Among the spectral traits, GNDVI provided the best accuracy for both yield and GPC prediction, with *R*^2^ (rRMSE) values of 0.785 (22.47%) and 0.687 (11.12%), respectively. Among the structural traits, Hmean performed the best for both yield and GPC prediction, with *R*^2^ (rRMSE) values of 0.787 (22.34%) and 0.783 (9.27%), respectively. Structural traits except for volume all showed high accuracy in the prediction of wheat yield and GPC, while the prediction accuracy of spectral traits showed a gradient difference in that GNDVI was the best, followed by CVI, NCPI, and MCARI. In addition, the rRMSE of GPC was always lower than that of yield by at least 10%.

### 4.2. Data Fusion (Two-to-One Model) in Yield and GPC Prediction

**Figure 4:**
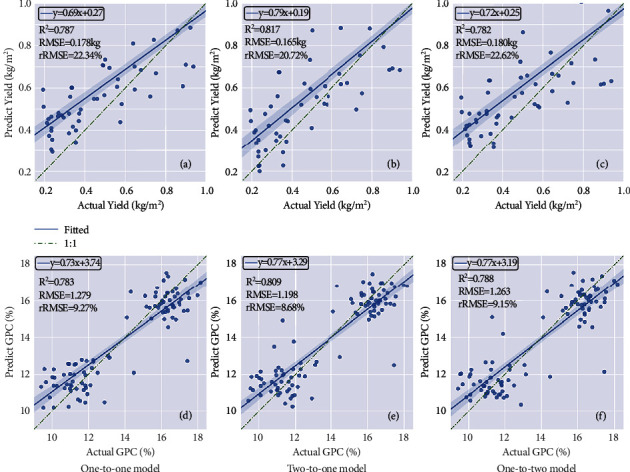
Scatter plots between the predicted value and ground truth. (a–c) Yield prediction results by the one-to-one model, two-to-one model, and one-to-two models; (d– f) GPC prediction results by the one-to-one, two-to-one model, and one-to-two models. The green dotted line represents the 1 : 1 fitted line.

To verify the effectiveness of data fusion, the two-to-one model is constructed and compared with the best one-to-one model, which has an *R*^2^ of 0.787 for yield and 0.783 for GPC prediction (Figures 4(a) and 4(d)). The best spectral feature (i.e., GNDVI) and the best structural feature (i.e., Hmean) were selected as two inputs to train the yield prediction and GPC prediction models, respectively. Compared to the best one-to-one model, the two-to-one model performed better for both yield (*R*^2^ = 0.817) and GPC (*R*^2^ = 0.809) prediction (Figures 4(b) and 4(e)).

### 4.3. Multitask Learning (One-to-Two Model) in Yield and GPC Prediction

To verify the effectiveness of multitask learning, the one-to-two model is constructed and compared with the best one-to-one model built with the Hmean feature. For multitask learning, the best feature (i.e., Hmean) was selected as input to the one-to-two model, which predicted yield and GPC simultaneously. The results showed that the one-to-two model provided high accuracy for both yield and GPC with *R*^2^ values of 0.782 and 0.788, respectively (Figures 4(c) and 4(f)), which is similar to the best one-to-one model (Figures 4(a) and 4(d)).

### 4.4. Data Fusion and Multitask Integration (Two-to-Two Model) in Yield and GPC Prediction

**Figure 5:**
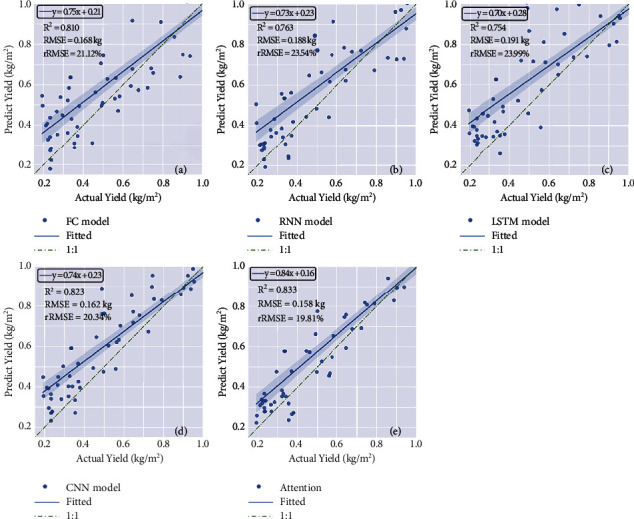
Scatter plots between the predicted and actual yield using two-to-two models with different feature extraction methods, including (a) FC, (b) RNN, (c) LSTM, (d) 1D CNN, and (e) attention model.

**Figure 6:**
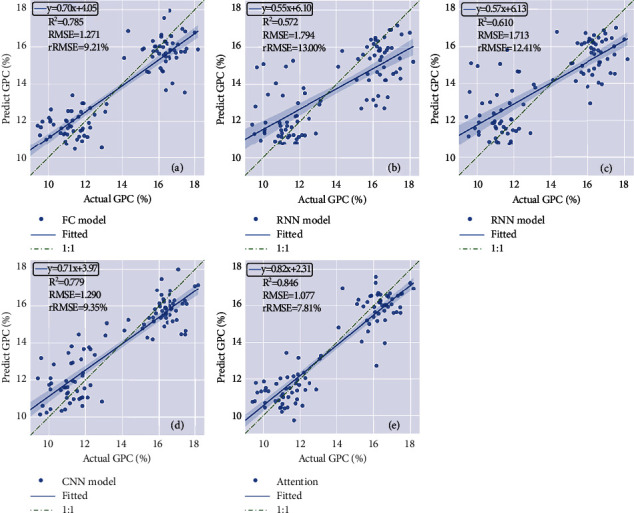
Scatter plots between the predicted and actual GPC using two-to-two models with different feature extraction methods, including (a) FC, (b) RNN, (c) LSTM, (d) 1D CNN, and (e) attention model.

To verify the effectiveness of data fusion and multitask integration, the FC two-to-two model is constructed and compared with the best one-to-one model. The GNDVI and Hmean were two inputs, and all the hidden layers were fully connected. The accuracies (*R*^2^) for yield and GPC were 0.810 and 0.785, respectively (Figure 5(a)and Figure 6(a)).

### 4.5. Influences of Feature Extraction on Two-to-Two Model

To further improve the prediction accuracy of the two-to-two model, RNN, LSTM, 1D CNN, and attention mechanism layers were selected to extract time-series features. The results showed that the yield prediction accuracies using different feature extraction methods were attention mechanism layer >1D CNN > FC > LSTM > RNN. Compared with FC, the RNN and LSTM methods did not improve the model accuracy. In contrast, the CNN model provided better accuracy than FC in terms of the mean yield *R*^2^ (0.823) and GPC *R*^2^ (0.779) (Figure 5(d) and Figure 6(d)). Additionally, the attention model provided the best accuracy for both yield and GPC prediction, with *R*^2^ values of 0.833 and 0.846, respectively (Figure 5(e) and Figure 6(e)).

## 5. Discussion

### 5.1. Performance of Different Features to Deep Learning-Based Yield/GPC Prediction (One-to-One Model)

In this study, all structural features, except volume, showed high accuracy in the prediction of wheat yield and GPC, outperforming most multispectral features. The importance of structural traits was consistent with previous findings in biomass estimation using LiDAR-derived traits [26]. They proved that the most important traits were height-related traits followed by PLA, and volume was even worse. The reason why volume is worse may be that it is a complicated 3D trait influenced easily by species and growth stages. In contrast, only the accuracy predicted by GNDVI spectral traits reached a high level similar to that of structural traits. The strength of GNDVI for yield prediction has also been proven by Shanahan et al. [32]. Because of the prediction differences of different traits, many studies have explored the best traits for modeling. This study selected 8 potential traits for modeling based on previous findings [32, 37]. Although the performance of each trait in yield (GPC) prediction is compared, the main purpose, unlike previous studies, is to select the best structural and spectral traits for comparative analysis of data fusion and multitask learning.

### 5.2. The Influence of Data Fusion on Deep Learning-Based Yield/GPC Prediction (Two-to-One Model)

The two-to-one model performed better than the one-to-one model for both yield (*R*^2^ = 0.817) and GPC (*R*^2^ = 0.809) prediction, which is consistent with previous data fusion studies using machine learning [13, 38]. The reasons may be two aspects. First, the information of multisource data is complementary in different periods, especially the multispectral and LiDAR data used in this study. LiDAR can monitor structural dynamics that reflect plant growth [28]. Spectral information can well characterize optical property changes caused by inner physiological statuses, especially in the development stage [39]. Second, data-driven deep learning models tend to have better performance with more features [13], which may not be suitable for traditional machine learning methods.

### 5.3. The Influence of Multitask Learning on Deep Learning-Based Yield/GPC Prediction (One-to-Two Model)

Multitask deep learning is conducive to sharing representations learned by different prediction tasks [20]. Because of the compound loss function of the one-to-two model, the influences of ratio change on prediction accuracy were analyzed using the one-to-two model. The yield/GPC weight ratios were changed from 0.1 : 0.9 to 0.9 : 0.1 (Table 2). The results showed that the prediction accuracy of GPC increases with the yield/GPC ratio. When the yield/GPC ratio was 0.1 : 0.9, the accuracy of GPC reached the maximum (*R*^2^ = 0.805, RMSE = 1.211, rRMSE =8.78%). In contrast, the accuracy of yield prediction reached the maximum (*R*^2^ = 0.799, RMSE = 0.173, rRMSE = 21.72%) when the yield/GPC ratio was 0.9 : 0.1. These results indicate that the priority of yield/GPC prediction accuracy could be controlled by adjusting the weight ratio between yield and GPC.

Multitask learning has been applied to learn the spatial features to improve the yield estimation accuracy of corn [23]. Although multitask learning did not significantly improve model accuracy in our study, it greatly optimized model efficiency because only one forward propagation was needed to predict both yield and GPC. In this study, wheat yield and GPC were equally critical target traits in breeding and cultivation, so the default weights of yield and GPC loss were set the same. Specifically, the default *λ*1 and *λ*2 both have normalized values of 0.5, considering the sum of *λ*1 and *λ*2 should be 1 for better training. Meanwhile, because the prediction accuracy of yield and GPC was adjustable in multitask learning (Table.  2), the model could be deployed for specific scenarios.

### 5.4. The Influence of Feature Extraction on Deep Learning-Based Yield/GPC Prediction (Two-to-Two Model)

The accuracy ranking of different feature extraction methods was attention mechanism layer >1D CNN > FC > LSTM > RNN. Unlike the advancement of LSTM and RNN performed in previous studies [40], they did not improve the model accuracy compared with FC in our study. In contrast, CNN showed better accuracy than FC, which suggested 1D CNN could solve some time-series problems with the advantage of capturing features of the local receptive field [41]. In yield and GPC prediction, the success of 1D CNN might be attributed to the extracted local features that correspond to key growth stages [20]. Moreover, the attention model provided the best prediction accuracy. This may be because the attention feature has a global receptive field that can take advantage of the temporal importance of different growth stages [23]. The attention feature also benefits the interpretation of time-series data contributions.

### 5.5. Interpretation of Time-Series Feature Importance

To understand the contribution of time-series data to the prediction accuracy of deep learning models, this study analyzed the daily contributions of structural and spectral properties based on the best two-to-two attention model (Figure S2(h)). The inputs of the model are GNDVI and Hmean, which are separately sent into the attention layer to extract the temporal attention features. The length of the attention feature vector is equal to the number of days, and the feature value represents the importance of each day.

**Figure 7:**
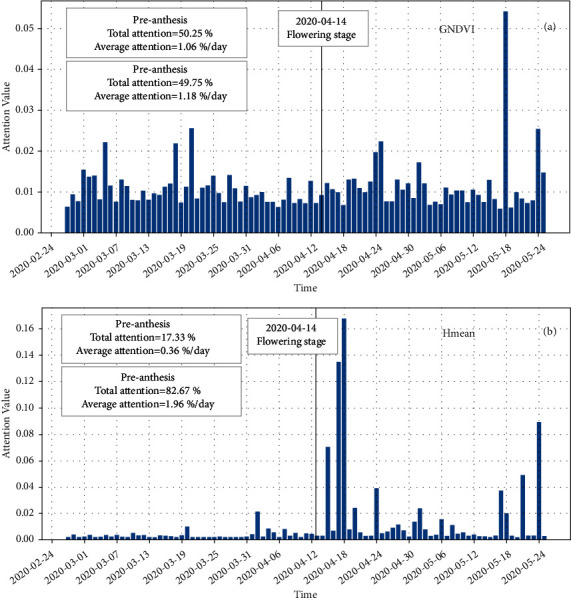
Bar plots of daily attention values of (a) GNDVI and (b) Hmean from feature attention analysis along the temporal dimension.

The two-to-two attention model captured different temporal patterns between GNDVI and Hmean. For GNDVI (Figure 7(a)), attention values were almost distributed on average, ranging from 0.5% to 3%. For Hmean (Figure 7(b)), two peaks appeared at the initial filling stage and the late mature stage, and the maximum value was at the initial filling stage. In addition, the average attention value preanthesis (1.06%/day) was lower than that postanthesis (1.18%/day) in the GNDVI. This trend was consistent but more pronounced in Hmean, which had average attention values of 0.36%/day and 1.96%/day preanthesis and postanthesis, respectively. The visualized attention pattern shows that the contribution of traits to yield and GPC prediction after anthesis is larger than that before anthesis, which was consistent with the results of studies on rice and soybean [42, 43].

### 5.6. Contributions and Future Works

This study explored the potential of simultaneous yield and GPC prediction with time-series data, utilizing many deep learning strategies. Specifically, we highlighted the following contributions: (1) multimodal and time-series data fusion significantly improved yield and GPC prediction accuracy; (2) Multitask learning was an efficient strategy for simultaneous yield and GPC prediction, and the priority of yield and GPC prediction accuracy could be controlled by adjusting the weight ratio; (3) a two-to-two model was proposed to integrate the mentioned multitask learning and time-series data fusion, while we also uncovered that the attention model provided the best prediction accuracy compared to FC, RNN, LSTM, and CNN; and (4) We deciphered that although the temporal patterns of structural traits and spectral traits were inconsistent, postanthesis was always a more important growth stage from the daily attention features.

In addition to the analysis results presented in this study, there are still some perspectives that need to be considered in the future: (1) This study selected some well-known traits for deep learning. Although selecting optimal traits for prediction is out of the study aims, it should be an important direction to automatically extract features for deep learning from raw imagery or point cloud data. (2) This study only analyzed the data fusion of spectral traits and structural traits from LiDAR. It is worth exploring more traits from more source data, such as thermal or even meteorological data [23]. In addition, data fusion is conducted at the feature level, and we acknowledge that fusion at raw data and decision levels is also interesting [13]. (3) This study adopted a simple but mature architecture for analysis. We suggest that future works consider some novel architectures, such as the Transformer, a new self-attention deep learning structure that can be trained in parallel with a good global receptive field. (4) We recognize the high cost of obtaining daily data. Therefore, developing low-cost platforms and exploring data generation methods (e.g, Generative Adversarial Networks) are encouraged to generate time-series and low-cost data in the future [44].

## 6. Conclusion

In this study, wheat yield and GPC were simultaneously predicted based on multitask learning from time-series proximal sensing. The results highlighted that the accuracy of both yield and GPC from multitask learning is comparable to the models that specifically predict yield or GPC. The accuracy improvement of data fusion to yield and GPC prediction is more obvious. Among the different time-series feature extraction methods, the attention mechanism layer outperformed CNN, FC, LSTM, and RNN. The daily importance revealed by attention features shows that both structural and spectral traits are more important after anthesis. In summary, this study implemented a systematic influence analysis of data fusion, feature extraction modules, and multitask learning on the accuracy of deep learning-based yield and GPC prediction. These findings may improve the understanding and application of deep learning-based prediction for yield and GPC in smart agriculture.

## Figures and Tables

**Table 1:** The description of the dataset.

Dataset	Number of examples	Number of features	Temporal dimension of each feature
Original	480	8	51 or 53

After interpolation and augmentation	1000	8	89

**Table 2:** Performance of yield and GPC prediction under different yield/GPC weight ratios.

Weight ratio (yield/GPC)	Yield			GPC		
	*R* ^2^	RMSE (kg)	rRMSE (%)	*R* ^2^	RMSE (%)	rRMSE (%)
0.9 : 0.1	0.799	0.173	21.72	0.755	1.357	9.83
0.8 : 0.2	0.795	0.175	21.93	0.754	1.360	9.86
0.7 : 0.3	0.794	0.175	21.95	0.776	1.329	9.41
0.6 : 0.4	0.790	0.177	22.17	0.754	1.361	9.86
0.5 : 0.5	0.782	0.180	22.62	0.788	1.263	9.15
0.4 : 0.6	0.780	0.181	22.69	0.801	1.225	8.87
0.3 : 0.7	0.766	0.187	23.39	0.765	1.327	9.63
0.2 : 0.8	0.770	0.185	23.20	0.786	1.268	9.19
0.1 : 0.9	0.752	0.192	24.11	0.805	1.211	8.78

## Data Availability

The datasets, source code, and other supporting data are available on request.

## Additional Points

*Highlights.* (i) Fusing multispectral and LiDAR data significantly improved the prediction accuracy of yield and grain protein content. (ii) Multitask deep learning enables the simultaneous prediction of yield and protein, and the accuracy is similar to that of the single-task yield or GPC prediction model. (iii) The attention-based deep learning model was better than other nonattention models in time-series feature extraction, which can also quantify the daily importance of features

## Supplementary Materials

Supplementary Materials Table S1: definitions and equations of the traits used in this study. Figure S1: the time curve graph of the four multispectral-derived spectral traits and the four LiDAR-derived structural traits. Figure S2: the details of different model architectures. Figure S3: the training process of the (a) one-to-one model; (b) one-to-two model; (c) two-to-one model; (d) two-to-two-FC model; (e) two-to-two-RNN model; (f) two-to-two-LSTM model; (g) two-to-two-CNN model; and (d) two-to-two-attention model. Table S2: detailed temporal information of data collection. Note: Repeat 1 and Repeat 2 are two biological replications. Cells filled with “Yes” and “No” represent valid and no observation, respectively.

## References

[1] Cantelaube P., Terres J. M. (2005). Seasonal weather forecasts for crop yield modelling in Europe. *Tellus Series a-Dynamic Meteorology and Oceanography*.

[2] Voon T. J., Edwards G. W. (1992). Research payoff from quality improvement: the case of protein in Australian wheat. *American Journal of Agricultural Economics*.

[3] Ma M. M., Li Y. C., Xue C. (2021). Current situation and key parameters for improving wheat quality in China. *Frontiers in Plant Science*.

[4] Schut A. G. T., Traore P. C. S., Blaes X., de By R. A. (2018). Assessing yield and fertilizer response in heterogeneous smallholder fields with UAVs and satellites. *Field Crops Research*.

[5] Chlingaryan A., Sukkarieh S., Whelan B. (2018). Machine learning approaches for crop yield prediction and nitrogen status estimation in precision agriculture: a review. *Computers and Electronics in Agriculture*.

[6] Yu N., Li L. J., Schmitz N., Tiaz L. F., Greenberg J. A., Diers B. W. (2016). Development of methods to improve soybean yield estimation and predict plant maturity with an unmanned aerial vehicle based platform. *Remote Sensing of Environment*.

[7] Wang Z., Chen J., Zhang J. (2021). Predicting grain yield and protein content using canopy reflectance in maize grown under different water and nitrogen levels. *Field Crops Research*.

[8] Wang L., Tian Y., Yao X., Zhu Y., Cao W. (2014). Predicting grain yield and protein content in wheat by fusing multi-sensor and multi-temporal remote-sensing images. *Field Crops Research*.

[9] Li B., Xu X., Zhang L. (2020). Above-ground biomass estimation and yield prediction in potato by using UAV- based RGB and hyperspectral imaging. *ISPRS Journal of Photogrammetry and Remote Sensing*.

[10] Li Z., Taylor J., Yang H. (2020). A hierarchical interannual wheat yield and grain protein prediction model using spectral vegetative indices and meteorological data. *Field Crops Research*.

[11] Barmeier G., Hofer K., Schmidhalter U. (2017). Mid-season prediction of grain yield and protein content of spring barley cultivars using high-throughput spectral sensing. *European Journal of Agronomy*.

[12] Pleijel H., Uddling J. (2011). Yield vs. quality trade-offs for wheat in response to carbon dioxide and ozone. *Global Change Biology*.

[13] Maimaitijiang M., Sagan V., Sidike P., Hartling S., Esposito F., Fritschi F. B. (2020). Soybean yield prediction from UAV using multimodal data fusion and deep learning. *Remote Sensing of Environment*.

[14] Ashapure A., Jung J. H., Chang A. J. (2020). Developing a machine learning based cotton yield estimation framework using multi-temporal UAS data. *ISPRS Journal of Photogrammetry and Remote Sensing*.

[15] Xue L. H., Cao W. X., Yang L. Z. (2007). Predicting grain yield and protein content in winter wheat at different N supply levels using canopy reflectance spectra. *Pedosphere*.

[16] Guo Q. H., Jin S. C., Li M. (2020). Application of deep learning in ecological resource research: theories, methods, and challenges. *Science China-Earth Sciences*.

[17] Pound M. P., Atkinson J. A., Wells D. M., Pridmore T. P., French A. P. Deep learning for multi-task plant phenotyping.

[18] LeCun Y., Bengio Y., Hinton G. (2015). Deep learning. *Nature*.

[19] Abdalla A., Cen H., Wan L., Mehmood K., He Y. (2021). Nutrient status diagnosis of infield oilseed rape via deep learning-enabled dynamic model. *IEEE Transactions on Industrial Informatics*.

[20] Furbank R. T., Silva-Perez V., Evans J. R. (2021). Wheat physiology predictor: predicting physiological traits in wheat from hyperspectral reflectance measurements using deep learning. *Plant Methods*.

[21] Yuan Q. Q., Shen H. F., Li T. W. (2020). Deep learning in environmental remote sensing: achievements and challenges. *Remote Sensing of Environment*.

[22] Sandhu K. S., Lozada D. N., Zhang Z., Pumphrey M. O., Carter A. H. (2021). Deep learning for predicting complex traits in spring wheat breeding program. *Frontiers in Plant Science*.

[23] Lin T., Zhong R., Wang Y. (2020). DeepCropNet: a deep spatial-temporal learning framework for county-level corn yield estimation. *Environmental Research Letters*.

[24] Wang T., Ding N. P., Li L. L., Lyu X. D., Chai Q., Dou X. C. (2020). Simulating the impact of long-term fertilization on basic soil productivity in a rainfed winter wheat system. *Agronomy-Basel*.

[25] Vadez V., Kholova J., Hummel G., Zhokhavets U., Gupta S. K., Hash C. T. (2015). LeasyScan: a novel concept combining 3D imaging and lysimetry for high-throughput phenotyping of traits controlling plant water budget. *Journal of Experimental Botany*.

[26] Jin S. C., Su Y. J., Song S. L. (2020). Non-destructive estimation of field maize biomass using terrestrial lidar: an evaluation from plot level to individual leaf level. *Plant Methods*.

[27] Li Q., Jin S. C., Zang J. (2022). Deciphering the contribution of spectral and structural data to wheat yield estimation from proximal sensing. *The Crop Journal: Under Review*.

[28] Su Y. J., Wu F. F., Ao Z. R. (2019). Evaluating maize phenotype dynamics under drought stress using terrestrial lidar. *Plant Methods*.

[29] Vincini M., Frazzi E., D'Alessio P. (2008). A broad-band leaf chlorophyll vegetation index at the canopy scale. *Precision Agriculture*.

[30] Penuelas J., Gamon J. A., Fredeen A. L., Merino J., Field C. B. (1994). Reflectance indices associated with physiological changes in nitrogen- and water-limited sunflower leaves. *Remote Sensing of Environment*.

[31] Daughtry C. S. T., Walthall C. L., Kim M. S., de Colstoun E. B., McMurtrey J. E. (2000). Estimating corn leaf chlorophyll concentration from leaf and canopy reflectance. *Remote Sensing of Environment*.

[32] Shanahan J. F., Schepers J. S., Francis D. D. (2001). Use of remote-sensing imagery to estimate corn grain yield. *Agronomy Journal*.

[33] Lu N., Zhou J., Han Z. X. (2019). Improved estimation of aboveground biomass in wheat from RGB imagery and point cloud data acquired with a low-cost unmanned aerial vehicle system. *Plant Methods*.

[34] Walter J. D. C., Edwards J., McDonald G., Kuchel H. (2019). Estimating biomass and canopy height with LiDAR for field crop breeding. *Frontiers in Plant Science*.

[35] Chu Z., Yu J. (2020). An end-to-end model for rice yield prediction using deep learning fusion. *Computers and Electronics in Agriculture*.

[36] Garbin C., Zhu X., Marques O. (2020). Dropout vs. batch normalization: an empirical study of their impact to deep learning. *Multimedia Tools and Applications*.

[37] Eugenio F. C., Grohs M., Venancio L. P. (2020). Estimation of soybean yield from machine learning techniques and multispectral RPAS imagery. *Remote Sensing Applications: Society and Environment*.

[38] Herzig P., Borrmann P., Knauer U. (2021). Evaluation of RGB and multispectral unmanned aerial vehicle (UAV) imagery for high-throughput phenotyping and yield prediction in barley breeding. *Remote Sensing*.

[39] Hassan M. A., Yang M., Rasheed A. (2021). Quantifying senescence in bread wheat using multispectral imaging from an unmanned aerial vehicle and QTL mapping. *Plant Physiology*.

[40] Cho W., Kim S., Na M., Na I. (2021). Forecasting of tomato yields using attention-based LSTM network and ARMA model. *Electronics*.

[41] Cao J., Zhang Z., Luo Y. C. (2021). Wheat yield predictions at a county and field scale with deep learning, machine learning, and google earth engine. *European Journal of Agronomy*.

[42] Wan L., Cen H., Zhu J. (2020). Grain yield prediction of rice using multi-temporal UAV-based RGB and multispectral images and model transfer - a case study of small farmlands in the South of China. *Agricultural and Forest Meteorology*.

[43] Zhang X., Zhao J., Yang G. (2019). Establishment of plot-yield prediction models in soybean breeding programs using UAV-based hyperspectral remote sensing. *Remote Sensing*.

[44] Araus J. L., Cairns J. E. (2014). Field high-throughput phenotyping: the new crop breeding frontier. *Trends in Plant Science*.

