# Supplementary material for: Simultaneous Prediction of Wheat Yield and Grain Protein Content Using Multitask Deep Learning from Time-Series Proximal Sensing
Source: Plant Phenomics. 2022 Mar 29;2022:9757948. doi: 10.34133/2022/9757948 (PMC8988204; doi:10.34133/2022/9757948)
Supplement: Supplementary Materials — Table S1: definitions and equations of the traits used in this study. Figure S1: the time curve graph of the four multispectral-derived spectral traits and the four LiDAR-derived structural traits. Figure S2: the details of different model architectures. Figure S3: the training process of the (a) one-to-one model; (b) one-to-two model; (c) two-to-one model; (d) two-to-two-FC model; (e) two-to-two-RNN model; (f) two-to-two-LSTM model; (g) two-to-two-CNN model; and (d) two-to-two-attention model. Table S2: detailed temporal information of data collection. Note: Repeat 1 and Repeat 2 are two biological replications. Cells filled with “Yes” and “No” represent valid and no observation, respectively. [file 9757948.f1.zip › Supplementary files.docx]

**Supplementary files**

**Table S1.** Definitions and equations of the traits used in this study.

| Spectral traits | Abbreviations | Formulas | References |
| --- | --- | --- | --- |
| Green normalized difference vegetation index | GNDVI | $(NIR-GREEN)/(NIR+GREEN)$ | [Xue et al. (2007)](#_ENREF_41) |
| Normalized chlorophyll pigment vegetation index | NCPI | $\text{（RED-B}LUE\text{）}/(RED+BLUE)$ | [Penuelas et al. (1994)](#_ENREF_25) |
| Chlorophyll vegetation index | CVI | $NIR*RED/GREEN^{2}$ | [Vincini et al. (2008)](#_ENREF_34) |
| Modified chlorophyll absorption reflectance index | MCARI | $\frac{NIR}{RED}\times(NIR-RED)-0.2\times(NIR-GREEN)$ | [Daughtry et al. (2000)](#_ENREF_10) |

Note: *RED*, *GREEN*, *BLUE*, and *NIR* are red, green, blue, and the near-infrared band reflectance values from multispectral data.


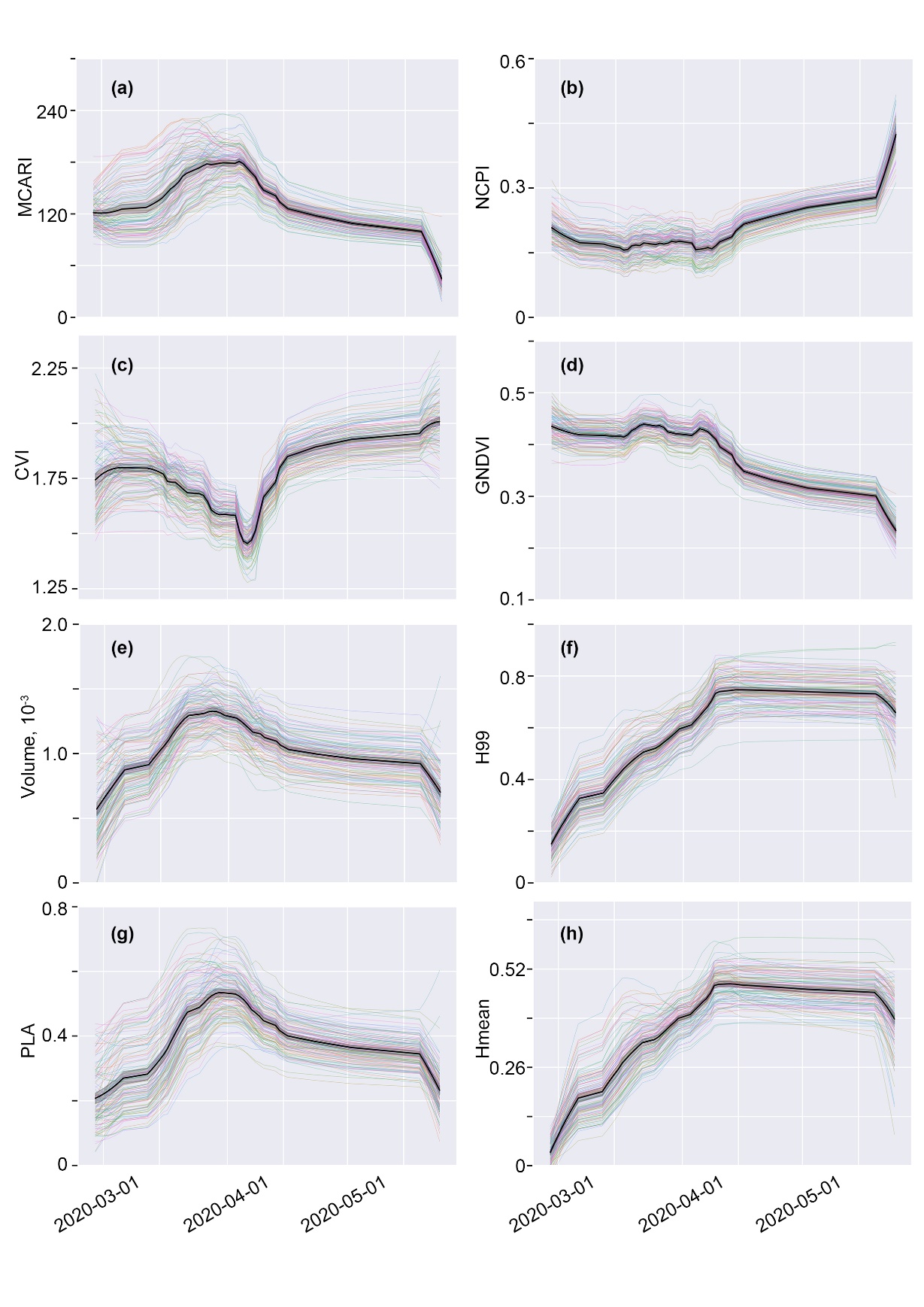


**Figure S1.** The time curve graph of the four multispectral-derived spectral traits and the four LiDAR-derived structural traits. (a) MCARI (Modified chlorophyll absorption reflectance index), (b) NCPI (Normalized chlorophyll pigment vegetation index), (c) CVI (Chlorophyll vegetation index), (d) GNDVI (Green normalized difference vegetation index), (e) Volume (plant volume), (f) H99 (99 percentile height of points), (g) PLA (projected leaf area), and (h) Hmean (mean height of points). In each subfigure, the colored lines represent the time curves of 120 varieties. Each colored line represents the mean value of two duplications of a variety under the control group. The black line represents the mean values (with 95% confidence interval) of all varieties.


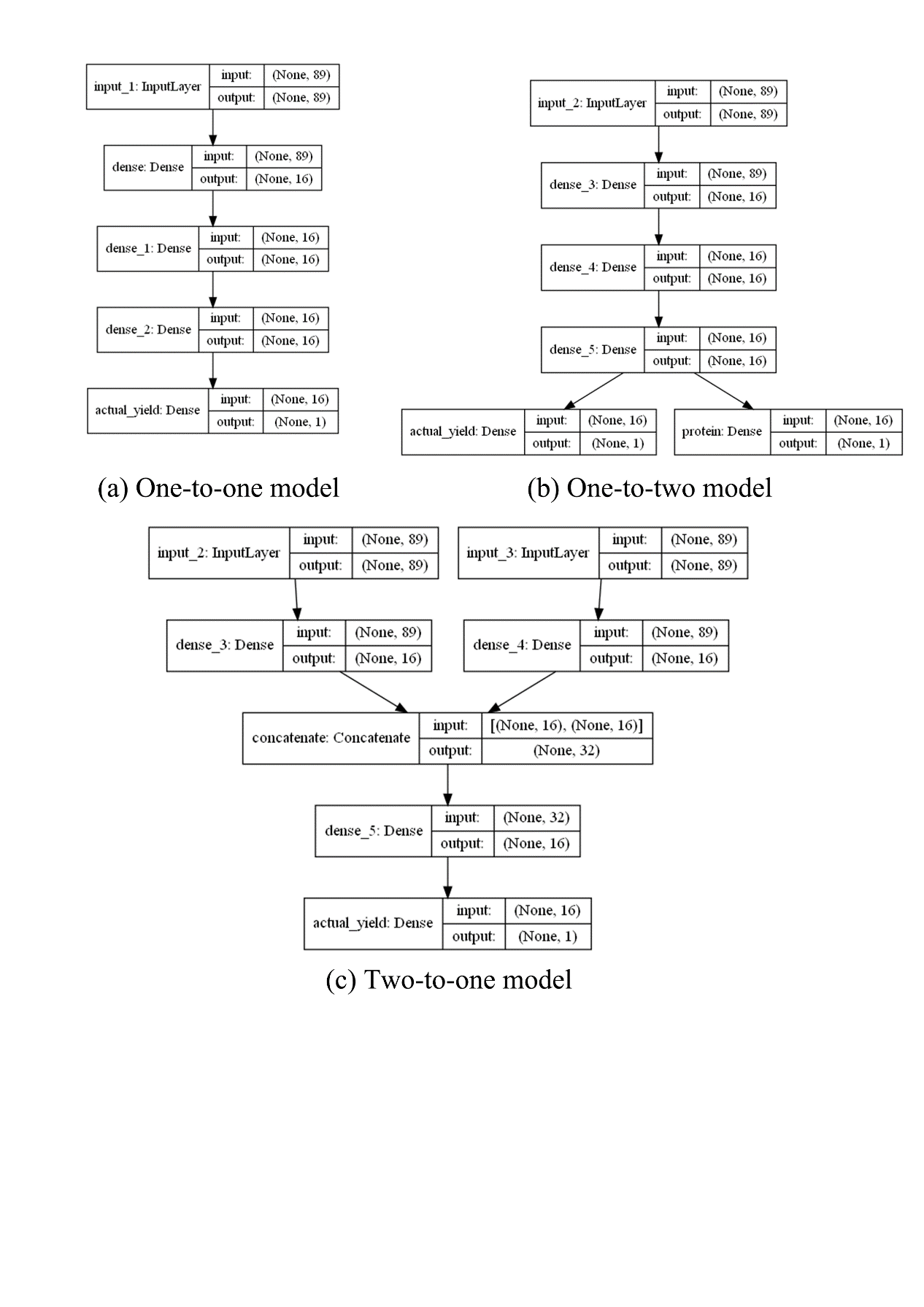


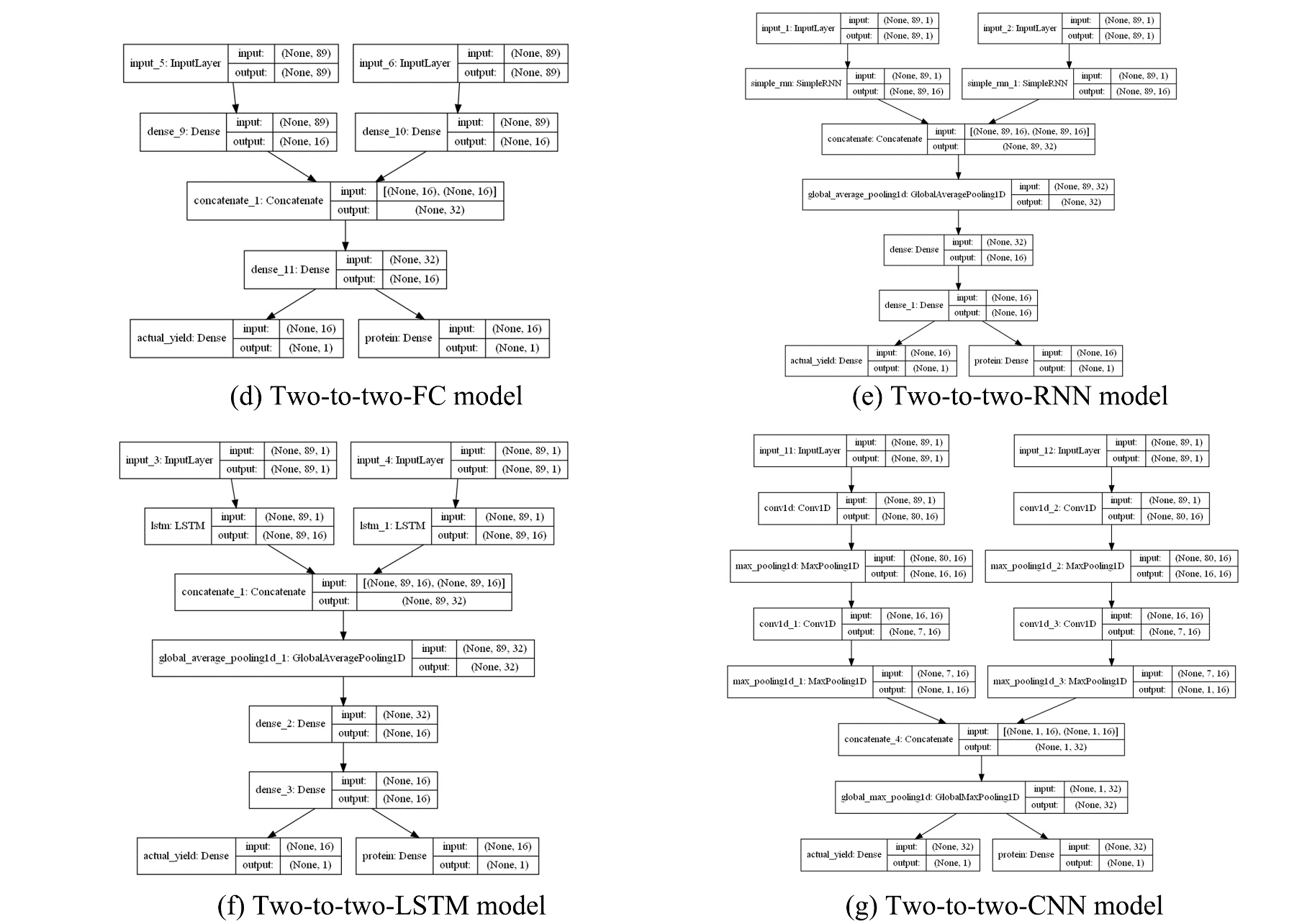


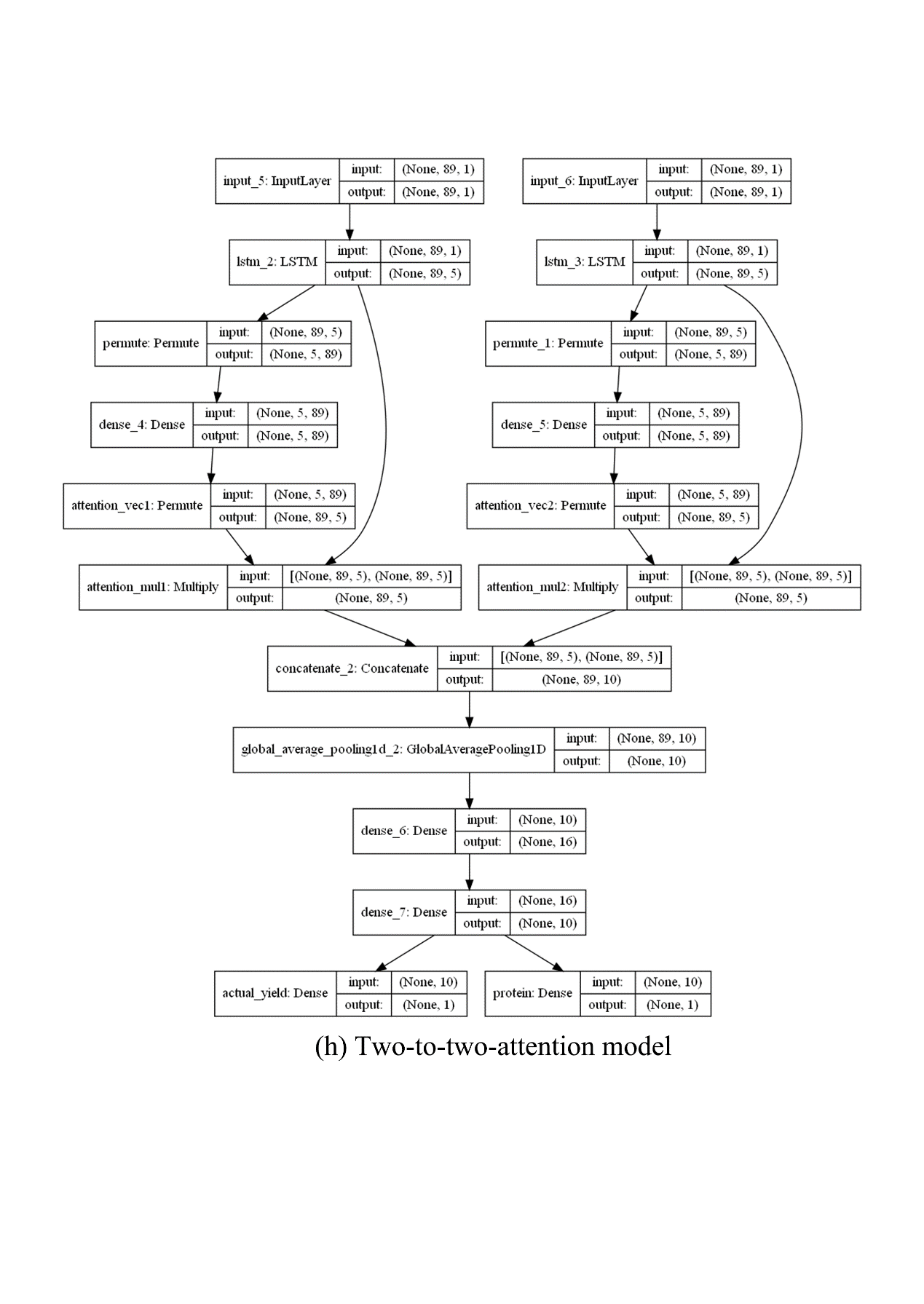


**Figure S2.** The details of different model architecture. (a) is the one-to-one model architecture; (b) is the one-to-two model architecture; c) is the two-to-one model architecture; (d) is the two-to-two-FC model architecture; (e) is the two-to-two-RNN model architecture; (f) is the two-to-two-LSTM model architecture; (g) is the two-to-two-CNN model architecture; (h) is the two-to-two-attention model architecture.


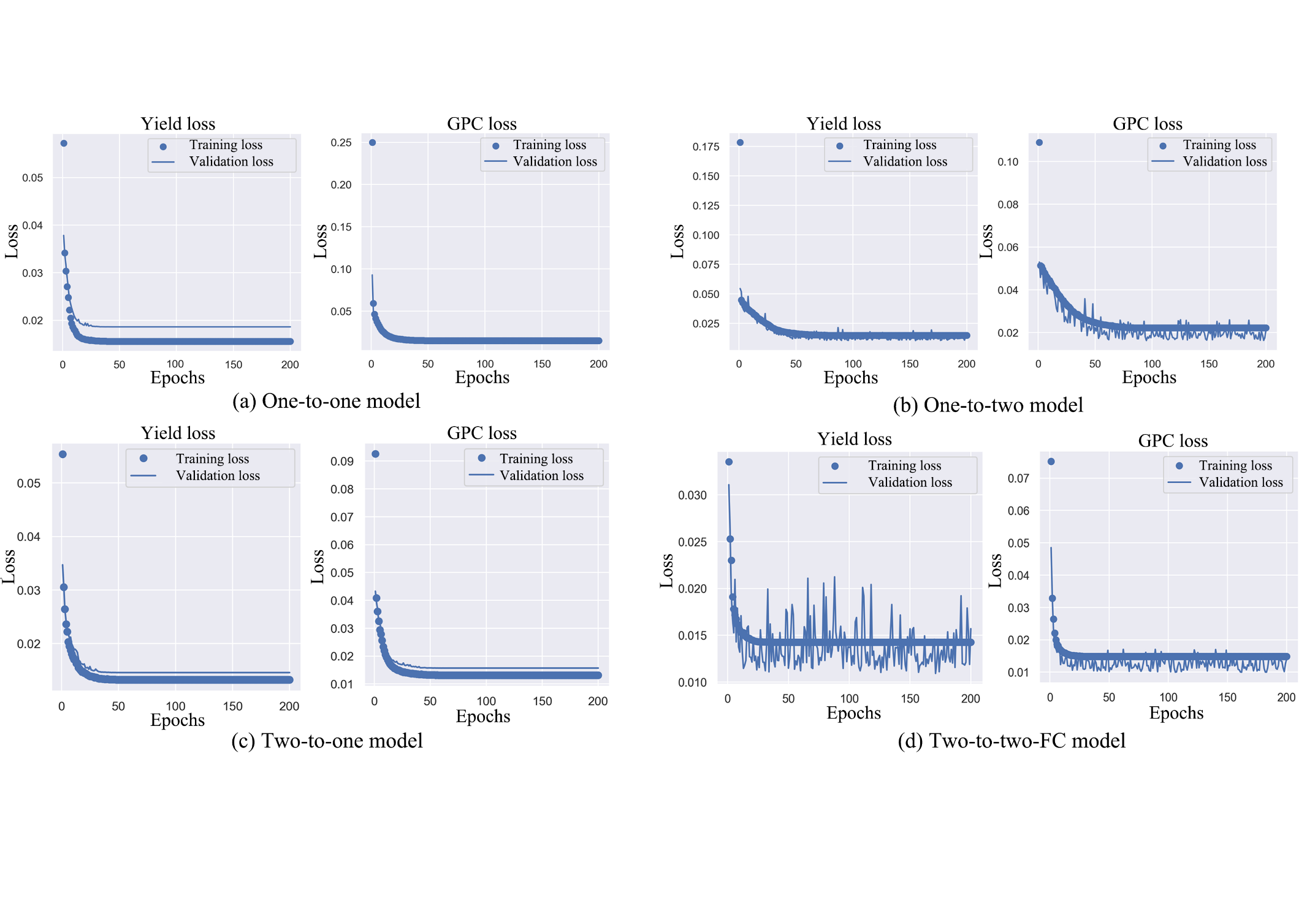


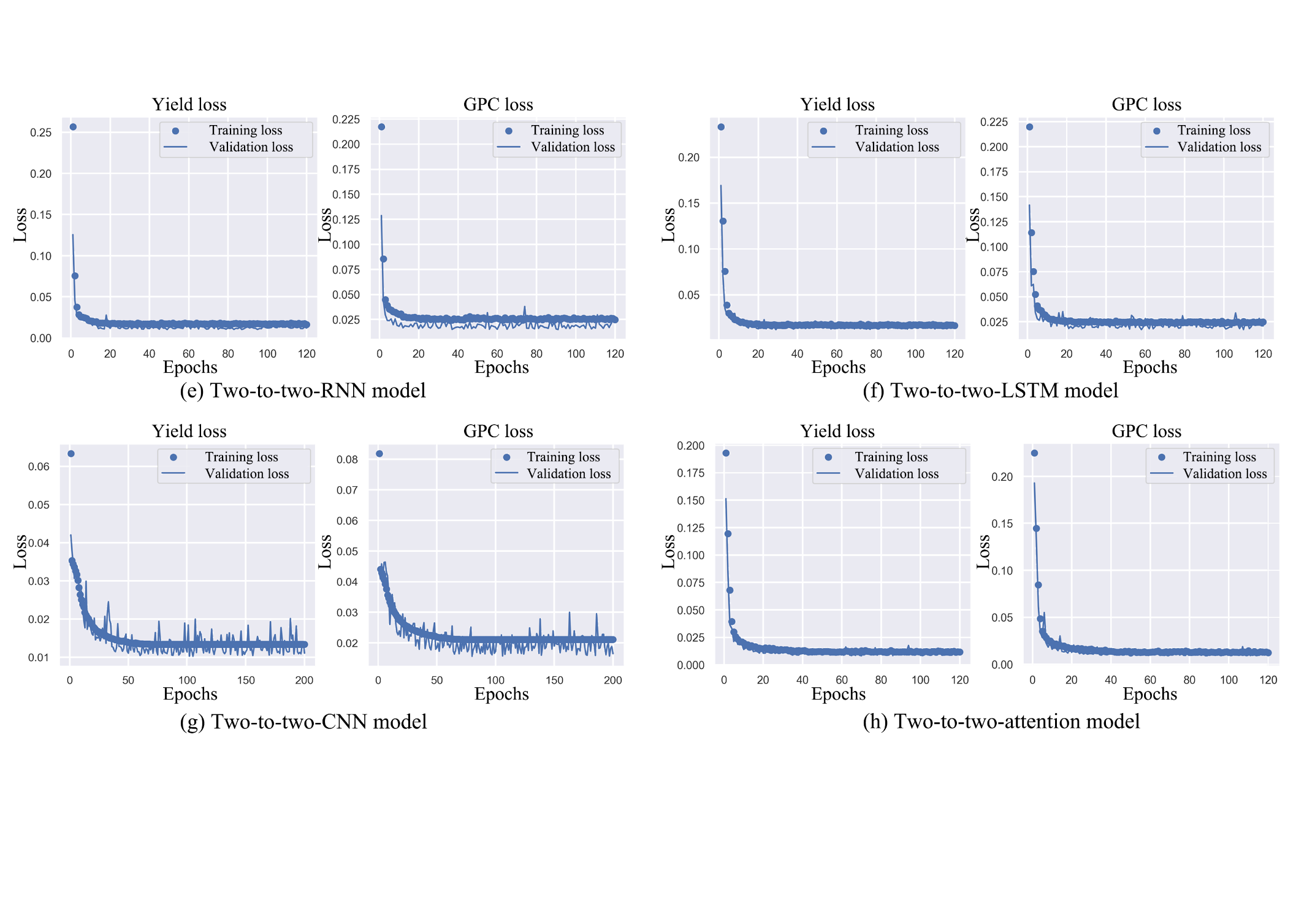


**Figure S3.** The training process of the (a) one-to-one model; (b) one-to-two model; (c) two-to-one model; (d) two-to-two-FC model; (e) two-to-two-RNN model; (f) two-to-two-LSTM model; (g) two-to-two-CNN model; and (d) two-to-two-attention model.
