# Supplementary material for: Simultaneous Prediction of Wheat Yield and Grain Protein Content Using Multitask Deep Learning from Time-Series Proximal Sensing
Source: Plant Phenomics. 2022 Mar 29;2022:9757948. doi: 10.34133/2022/9757948 (PMC8988204; doi:10.34133/2022/9757948)
Supplement: Supplementary Materials — Table S1: definitions and equations of the traits used in this study. Figure S1: the time curve graph of the four multispectral-derived spectral traits and the four LiDAR-derived structural traits. Figure S2: the details of different model architectures. Figure S3: the training process of the (a) one-to-one model; (b) one-to-two model; (c) two-to-one model; (d) two-to-two-FC model; (e) two-to-two-RNN model; (f) two-to-two-LSTM model; (g) two-to-two-CNN model; and (d) two-to-two-attention model. Table S2: detailed temporal information of data collection. Note: Repeat 1 and Repeat 2 are two biological replications. Cells filled with “Yes” and “No” represent valid and no observation, respectively. [file 9757948.f1.zip › Supplementary Table S2.docx]

**Table S2.** Detailed temporal information of data collection. Note: Repeat 1 and Repeat 2 are two biological replications. Cells filled with "Yes" and “No” represent valid and no observation, respectively.

| **Date** | **Days after sowing** | **Repeat 1** | **Repeat 2** |
| --- | --- | --- | --- |
| 2/27/2020 | 107 | Yes | Yes |
| 2/28/2020 | 108 | Yes | Yes |
| 2/29/2020 | 109 | Yes | Yes |
| 3/1/2020 | 110 | Yes | Yes |
| 3/2/2020 | 111 | Yes | Yes |
| 3/3/2020 | 112 | Yes | Yes |
| 3/4/2020 | 113 | Yes | Yes |
| 3/5/2020 | 114 | Yes | Yes |
| 3/6/2020 | 115 | Yes | No |
| 3/7/2020 | 116 | No | No |
| 3/8/2020 | 117 | No | No |
| 3/9/2020 | 118 | No | No |
| 3/10/2020 | 119 | No | No |
| 3/11/2020 | 120 | No | No |
| 3/12/2020 | 121 | Yes | Yes |
| 3/13/2020 | 122 | Yes | Yes |
| 3/14/2020 | 123 | Yes | Yes |
| 3/15/2020 | 124 | Yes | Yes |
| 3/16/2020 | 125 | Yes | Yes |
| 3/17/2020 | 126 | Yes | Yes |
| 3/18/2020 | 127 | Yes | Yes |
| 3/19/2020 | 128 | Yes | Yes |
| 3/20/2020 | 129 | Yes | Yes |
| 3/21/2020 | 130 | Yes | Yes |
| 3/22/2020 | 131 | Yes | No |
| 3/23/2020 | 132 | No | No |
| 3/24/2020 | 133 | No | No |
| 3/25/2020 | 134 | Yes | Yes |
| 3/26/2020 | 135 | Yes | Yes |
| 3/27/2020 | 136 | Yes | Yes |
| 3/28/2020 | 137 | Yes | Yes |
| 3/29/2020 | 138 | Yes | Yes |
| 3/30/2020 | 139 | Yes | Yes |
| 3/31/2020 | 140 | Yes | Yes |
| 4/1/2020 | 141 | No | No |
| 4/2/2020 | 142 | No | No |
| 4/3/2020 | 143 | Yes | Yes |
| 4/4/2020 | 144 | Yes | Yes |
| 4/5/2020 | 145 | Yes | Yes |
| 4/6/2020 | 146 | Yes | Yes |
| 4/7/2020 | 147 | Yes | Yes |
| 4/8/2020 | 148 | Yes | Yes |
| 4/9/2020 | 149 | Yes | Yes |
| 4/10/2020 | 150 | Yes | Yes |
| 4/11/2020 | 151 | Yes | Yes |
| 4/12/2020 | 152 | No | No |
| 4/13/2020 | 153 | Yes | Yes |
| 4/14/2020 | 154 | Yes | Yes |
| 4/15/2020 | 155 | Yes | Yes |
| 4/16/2020 | 156 | Yes | Yes |
| 4/17/2020 | 157 | Yes | Yes |
| 4/18/2020 | 158 | Yes | Yes |
| 4/19/2020 | 159 | No | No |
| 4/20/2020 | 160 | No | No |
| 4/21/2020 | 161 | No | No |
| 4/22/2020 | 162 | Yes | Yes |
| 4/23/2020 | 163 | Yes | Yes |
| 4/24/2020 | 164 | No | No |
| 4/25/2020 | 165 | No | No |
| 4/26/2020 | 166 | No | No |
| 4/27/2020 | 167 | No | No |
| 4/28/2020 | 168 | No | No |
| 4/29/2020 | 169 | No | No |
| 4/30/2020 | 170 | No | No |
| 5/1/2020 | 171 | No | No |
| 5/2/2020 | 172 | Yes | Yes |
| 5/3/2020 | 173 | Yes | Yes |
| 5/4/2020 | 174 | No | No |
| 5/5/2020 | 175 | No | No |
| 5/6/2020 | 176 | No | No |
| 5/7/2020 | 177 | No | No |
| 5/8/2020 | 178 | No | No |
| 5/9/2020 | 179 | No | No |
| 5/10/2020 | 180 | No | No |
| 5/11/2020 | 181 | No | No |
| 5/12/2020 | 182 | No | No |
| 5/13/2020 | 183 | No | No |
| 5/14/2020 | 184 | No | No |
| 5/15/2020 | 185 | No | No |
| 5/16/2020 | 186 | No | No |
| 5/17/2020 | 187 | No | No |
| 5/18/2020 | 188 | No | No |
| 5/19/2020 | 189 | Yes | Yes |
| 5/20/2020 | 190 | Yes | Yes |
| 5/21/2020 | 191 | Yes | Yes |
| 5/22/2020 | 192 | Yes | Yes |
| 5/23/2020 | 193 | Yes | Yes |
| 5/24/2020 | 194 | Yes | Yes |
| 5/25/2020 | 195 | Yes | Yes |
| **Valid observation days** | **89** | **53** | **51** |
